# Differential effects of the methylenetetrahydrofolate reductase polymorphisms (C677T and A1298C) on hematological malignancies among Latinos: a meta-analysis

**DOI:** 10.1590/1678-4685-GMB-2018-0161

**Published:** 2019-11-14

**Authors:** Samanta Celeste Garcia-Hernandez, Perla Meneses-Sanchez, Leonardo Martin Porchia, Enrique Torres-Rasgado, Ricardo Pérez-Fuentes, Martha Elba Gonzalez-Mejia

**Affiliations:** 1 Departamento de Genética, Facultad de Medicina, Benémerita Universidad Autónoma de Puebla. Puebla, Mexico.; 2 Laboratorio de Investigación en Fisiopatología de Enfermedades Crónicas, Centro de Investigación Biomédica de Oriente, IMSS, Delegación Puebla. Atlixco, Puebla, Mexico.; 3 Facultad de Medicina, Benemérita Universidad Autónoma de Puebla. Puebla, Mexico.

**Keywords:** MTHFR, protective factor, Latin America, leukemia

## Abstract

Our objective was to determine the association between the methylenetetrahydrofolate reductase polymorphisms (C677T and A1298C) and the risk of developing acute lymphoblastic leukemia (ALL), chronic myeloid leukemia (CML), acute myeloid leukemia (AML), and multiple myelomas (MM) in Latinos. PubMed, SCOPUS, EBSCO, LILACS, and other Latin-specific databases were searched for case-control studies that investigated the association between these polymorphisms and hematologic malignancies until November 2017. Genotype distributions were extracted and either fixed-effects or random-effects models were used to calculate the pooled crude odds ratios (ORs) for the heterozygous, homozygous, dominant, recessive, and allelic genetic models. No publication bias was detected by the Begg-Mazumdar’s test and Egger’s test. From 290 publications, we identified 15 studies on the C677T polymorphism and 13 studies on the A1298C polymorphism. We observed a significant decrease in risk for the C677T polymorphism (OR range=0.54-0.75, *p*<0.01) and a significant increase in risk for the A1298C polymorphism (OR range=1.28-2.52, *p*<0.05) in developing ALL for all genetic models. No associations were determined for CML, AML, or MM for either polymorphism. This meta-analysis demonstrated that the A1298C polymorphism was associated with an increased risk of developing ALL, whereas the C677T polymorphism was associated with a decreased risk (protective factor) in the Latino population.

## Introduction

Characterized as an uncontrolled growth of cells, cancer is a multi-stage and multi-factorial process (Mendis, 2014) with environmental factors, such as diet, lifestyle habits (Tomasetti and Vogelstein, 2015), and a genetic predisposition, conferring a strong individual risk. Methylenetetrahydrofolate reductase (MTHFR) has been recently reported to be associated with diet and cancer development (Xie *et al.*, 2014). With low folic acid consumption among Latin Americans (Brito *et al.*, 2015), MTHFR, a key metabolite of the folate metabolism pathway, presents as a specific node between diet and cancer development.

The *mthfr* gene is located on chromosome 1 and is a key enzyme for reducing 5,10-methylenetetrahydrofolate to 5-methyltetrahydrofolate (Crider *et al.*, 2012). Low levels of folate or defects in folate metabolism may increase the risk of DNA strand breaks, aberrant DNA methylation, or even deficiencies in the DNA repair process, all of which are associated with an increased risk of cancer development (Suzuki and Bird, 2008). Two of the most studied polymorphisms of MTHFR are C677T and A1298C. The C677T polymorphism is associated with a 66% and 25% decrease of enzymatic activity for the heterozygous and homozygous genotypes, respectively, whereas the A1298C polymorphism is associated with a less severe decrease of enzyme activity (Tang *et al.*, 2014).

Previous reports have established that the C677T and the A1298C polymorphisms are potential risk factors for the development of prostate, colon, and breast cancers in certain ethnicities (Yu and Chen, 2012; Rai, 2015; Zhu *et al.*, 2016). Even though inconsistencies remain for this relationship among many ethnicities, recent meta-analyses have been performed showing that these polymorphisms are associated with an increased risk of developing acute myeloid leukemia (AML) (Dong *et al.*, 2014) and multiple myelomas (MM) (Ma *et al.*, 2009)[Bibr B21], and a decreased risk of developing acute lymphoblastic leukemia (ALL) (Xie *et al.*, 2015) in Caucasians and Asians. However, there is a lack of consideration for the Latin America population. For example, Jiang *et al.* (2013) included Latinos in the “Others” category, which also consisted of studies from Turkey, Serbia, and Egypt (Jiang *et al.*, 2013, Li *et al.*, 2015a). With so many studies focusing on the C677T and A1298C polymorphisms and cancer susceptibility in the Latin American population yielding no concise result, the aim of this meta-analysis was to determine the effect of the C677T and A1298C polymorphisms on hematological malignancies development in Latinos.

## Methods

### Search strategy

This meta-analysis was perform according to the PRISMA guidelines (Moher *et al.*, 2010) (Table S1). PubMed, Wiley, SCOPUS, EBSCO, LILACS, BIBLAT, CABI, DOAJ, GALE, IMBIOMED, LATININDEX, MEDIGRAPHIC, PERIODICA, and REDALYC databases were searched for studies that investigated the association between the MTHFR polymorphisms and cancer in Latin Americans. The following keywords/terms and any of their derivations were used: “Latino or Hispanic” as well as other terms associated with Latin American countries, “MTHFR or methylenetetrahydrofolate”, “polymorphism or SNP”, and “cancer or carcinogenesis” (Table S2). Latin American countries were identified according to the United Nations Educational, Scientific and Cultural Organization and the Community of Latin American and Caribbean States (CELAC) definitions (NTI, 2011). However, studies taken in the USA or other parts of the world, where subjects identified themselves as Latin Americans, were also considered. Due to the significant heterogeneity of Latin Americans, studies that focused on Asians, Germans, or Jewish immigrants/descendants were not considered. The search was performed without any language restrictions for publications published until November 20, 2017. Afterwards, the complied publications references were hand searched.

### Inclusion and exclusion criteria

Two authors determined if a study should be included. If a disagreement occurred about a publication, a third author analyzed the publication in question. Initially, the titles and abstract were examined to determine if the article was original research that focused on hematologic malignancies (ALL, AML, CML, or MM), Latinos, and MTHFR. For inclusion, the studies must had met the following criteria: 1) case-controls studies; 2) examined at least one of the MTHFR polymorphisms (C677T or A1298C); 3) focused on human subjects that were Latinos or of Latino-descendants; 4) patients with a diagnosis that was confirmed by either pathological or histological examination; and 5) contained information about genotype frequencies. Studies were excluded if: 1) not a case-control study; 2) information was used in a previous publication; 3) failed to describe cancer conformation; 4) failed to report the complete genotype distribution or unable to determine it from the reported data; 5) failed to use local controls; or 6) were a meta-analysis, review, or editorial article.

### Bias analysis and data extraction

Two authors independently assessed the quality of the studies using the Newcastle-Ottawa Quality Assessment Scale (Stang, 2010). The following aspects of each study were appraised: selection of cases and controls, comparability, and outcome (Table S3, Figure S1). For analysis, the quality scores ranged from 0 to 9. Studies that scored ≥6 were considered of high quality. The following data was collected from each study: first author’s name, year of publication, geographical location, type of cancer, technique used to detect the polymorphism, source of controls, and the genotype distribution for cases and controls.

### Statistical analysis

For each study, the Hardy-Weinberg Equilibrium (HWE) was determined by the Ψ^2^-test for the controls and a *p*-value <0.05 was considered in agreement. Crude odds ratios (ORs) and 95% confidence intervals (95%CI) were used to assess the strength of the association between the MTHFR polymorphism and the risk of cancer. The pooled crude ORs were calculated for allelic (2 vs. 1), dominant (12 + 22 vs. 11), recessive (22 vs. 12 + 11), heterozygous (12 vs. 11), and homozygous (22 vs. 11) genetic models, where 1 corresponded to the wild-type and 2, the mutant form. Heterogeneity was determined using the Ψ^2^-based Q-test and its degree was assessed by the inconsistency index (I^2^). Depending on the results of heterogeneity tests, either the random effects model (Ψ^2^-based Q-test *p*<0.10 and I2>50%) (Miller, 1978) or fixed effects model (DerSimonian and Laird, 1986) was selected to calculate the pooled OR and 95%CI. Sensitivity analysis by removing one study and recalculating the pooled OR and 95%CI was conducted to verify the stability of the results. Begg’s funnel plot, Begg-Mazumdar’s test (Begg and Mazumdar, 1994), and Egger’s linear regression test (Egger *et al.*, 1997) were used to assess publication bias. All the statistical analyses were conducted by using Review Manager (RevMan) v5.3 (Copenhagen, DK) and StatDirect Statistical Software v2.8 (Cheshire, UK). Unless noted otherwise, *p*-values <0.05 (two-sided) were considered statistically significant.

## Results

### Eligible studies

**Figure 1:**
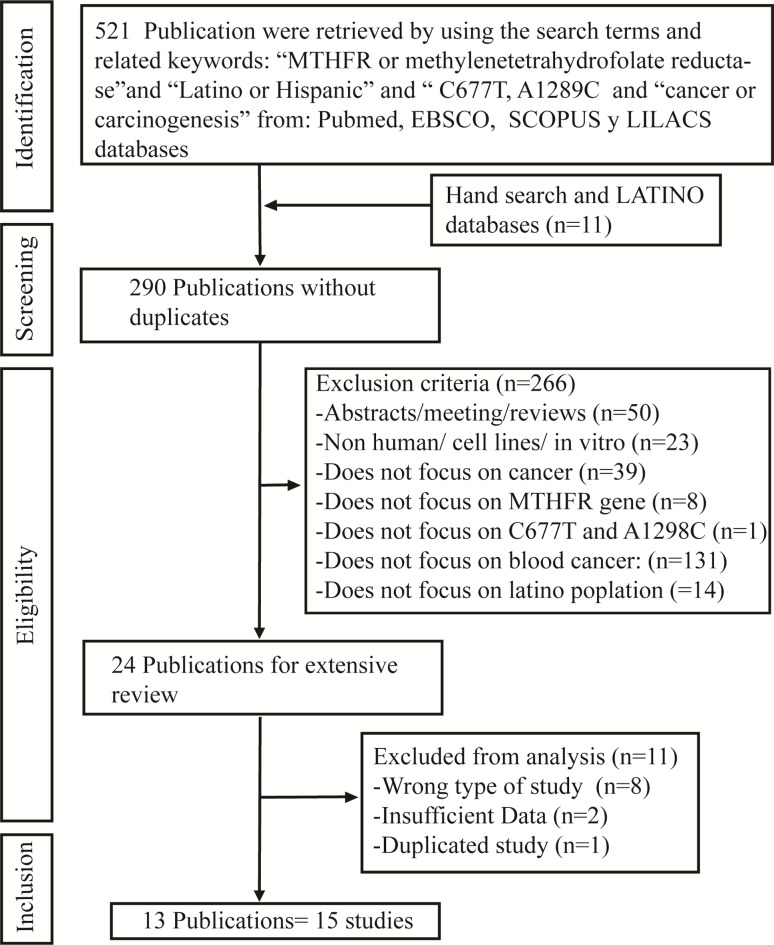
Flow chart for literature review of studies to be included in the meta-analysis.

A total of 521 publications were retrieved from searching multiple databases and reviewing the publications bibliographies (Figure 1); however, the cohort consisted of 290 publications after removing duplicate records. Two hundred and sixty-six publications were excluded because they were conference abstracts or reviews, focused on animals or cell lines, did not focus on the Latino population, were about non-hematologic cancers, or did not examine the MTHFR polymorphisms. The remaining 24 publications were extensively evaluated. Eight publications were not case-control studies, two publications lacked sufficient information, and one publication used previously published data; therefore, these 11 publications were excluded. This resulted in 13 publications (15 studies) that were included in this meta-analysis (Franco *et al.*, 2001; Zanrosso *et al.*, 2005, 2006; da Costa Ramos *et al.*, 2006; Ruiz-Argüelles *et al.*, 2007; Amorim *et al.*, 2008; Barbosa *et al.*, 2008; Gallegos-Arreola *et al.*, 2008; Lima *et al.*, 2008; Metayer *et al.*, 2011; Lordelo *et al.*, 2012; Silva *et al.*, 2013; Gutiérrez-Álvarez *et al.*, 2016), and three studies from Mexico (Ruiz-Argüelles *et al.*, 2007; Gallegos-Arreola *et al.*, 2008; Gutiérrez-Álvarez *et al.*, 2016). One study focused on Latinos living in the USA (Metayer *et al.*, 2011).

Four types of hematologic malignancies were assessed in this meta-analysis. The most representative hematologic malignancy was ALL with 7 studies (Franco *et al.*, 2001; Zanrosso *et al.*, 2006; Ruiz-Argüelles *et al.*, 2007; Gallegos-Arreola *et al.*, 2008; Metayer *et al.*, 2011; Silva *et al.*, 2013; Gutiérrez-Álvarez *et al.*, 2016) on C677T and five studies on A1298C (Franco *et al.*, 2001; Zanrosso *et al.*, 2006; Metayer *et al.*, 2011; Silva *et al.*, 2013; Gutiérrez-Álvarez *et al.*, 2016). AML had 5 studies for C677T and A1298C (Zanrosso *et al.*, 2005; da Costa Ramos *et al.*, 2006; Amorim *et al.*, 2008; Barbosa *et al.*, 2008; Silva *et al.*, 2013). Both polymorphisms were examined by the two studies on CML (Barbosa *et al.*, 2008; Lordelo *et al.*, 2012) and the only one study for MM (Lima *et al.*, 2008). The control genotype distribution for all the studies was consistent with HWE, except for one study (Lordelo *et al.*, 2012). Another study (Ruiz-Argüelles *et al.*, 2007) was found to contain a high level of bias (score<6) by the Ottawa-New Castle guidelines. The publication years of the involved studies ranged from 2001 to 2016. The characteristics of the included studies are summarized in Table 1.

**Table 1:** Characteristics of included studies.

Studies (Country)	Age (years)	Type of Cancer	SNP	Group	Genotype ^1^			HWE ^2^	Control ^3^	Score ^4^
					11	12	22			
Amorin, 2008 ^5^	Cases and controls:	AML	C677T	Controls	35	25	2	0.32	PB	7
(Brazil)	Identified as children			Cases	24	20	5			
			A1298C	Controls	40	16	4	0.19	PB	7
				Cases	30	14	6			
Barbosa, 2008	Cases: Median age = 27	AML	C677T	Controls	65	29	6	0.27	PB	6
(Brazil)	(Range: 6-70)			Cases	17	8	2			
	Controls: Median age = 29		A1298C	Controls	63	32	5	0.72	PB	6
	(Range 18-40)			Cases	15	11	1			
	Cases: Median age = 44	CML	C677T	Controls	65	29	6	0.27	PB	6
	(Range: 9-93)			Cases	46	19	2			
	Controls: Median age = 29 (Range: 18-40)		A1298C	Controls	63	32	5	0.72	PB	6
				Cases	41	23	3			
da Costa Ramos, 2006	Cases: Average age = 7.1 ± 5.8 Controls: Average age = 5.4 ± 5.2	AML	C677T	Controls	156	128	31	0.53	PB	7
(Brazil)				Cases	93	67	22			
			A1298C	Controls	190	104	21	0.20	PB	7
				Cases	104	62	16			
Franco, 2001	Cases and controls:	ALL	C677T	Controls	22	36	13	0.80	HB	8
(Brazil)	Mean age = 6-7			Cases	36	28	6			
	(Range: 0.2-15)		A1298C	Controls	41	28	2	0.27	HB	8
			Cases	36	30	5				
Gallegos-Arreola, 2008	Cases and controls:	ALL	C677T	Controls	59	79	32	0.54	PB	6
Average age = 40										
(Mexico)				Cases	64	78	28			
Gutierrez-Alvarez, 2016	Cases: Average age = 6.9	ALL	C677T	Controls	42	72	38	0.52	N/A	8
(Range 1-15)										
(Mexico)	Controls: Average age = 6.7			Cases	22	36	12			
			A1298C	Controls	108	42	2	0.35	N/A	8
				Cases	50	14	6			
Lima, 2008	Cases: Average age = 57.2 ± 11.4, Controls: Average age = 3.8 ± 2.9	MM	C677T	Controls	92	79	17	0.99	HB	6
(Brazil)				Cases	52	57	14			
			A1298C	Controls	127	49	12	0.02	HB	6
				Cases	79	33	11			
Lordelo, 2012	Cases and controls:	CML	C677T	Controls	140	114	19	0.52	PB	7
Identified as adults (≥20)										
(Brazil)				Cases	46	47	12			
			A1298C	Controls	119	143	11	<0.01 *	PB	7
				Cases	61	43	1			
Metayer, 2011	Cases and controls:	ALL	C677T	Controls	59	91	27	0.40	PB	8
(USA)	Identified as children (<15)			Cases	62	72	20			
			A1298C	Controls	110	62	6	0.44	PB	8
				Cases	86	60	8			
Ruiz-Arguelles, 2007	Cases: Median age =16	ALL	C677T	Controls	155	384	251	0.71	PB	4*
(Mexico)	(Range: 0-40)			Cases	2	10	16			
Controls: Not provided										
Silva, 2013	Cases and controls:	ALL	C677T	Controls	95	108	21	0.22	PB	7
(Brazil)	Identified as children (<19).			Cases	82	53	9			
			A1298C	Controls	147	82	19	0.12	PB	7
				Cases	55	53	28			
		AML	C677T	Controls	95	108	21	0.22	PB	7
				Cases	19	12	2			
			A1298C	Controls	147	82	19	0.12	PB	7
				Cases	13	13	5			
Zanrosso 2005	Cases: Median age = 4	AML	C677T	Controls	123	95	22	0.56	PB	8
(Brazil)	(Range: 0-16)			Cases	21	17	5			
Controls: Median age = 3.5										
			A1298C	Controls	151	77	18	0.07	PB	8
				Cases	28	13	1			
Zanrosso 2006	Cases: Average age = 6.2	ALL	C677T	Controls	96	82	20	0.69	PB	6
(Brazil)	Controls: Average age = 25			Cases	96	56	13			
			A1298C	Controls	111	76	12	0.83	PB	6
				Cases	83	74	11			

1 11, 12, and 22 indicates the frequency of the wild-type, heterozygote, and homozygote mutant, respectively, where 1 is the C-allele for the C677T polymorphism and A-allele for the A1298C polymorphism, and 2 is the T-allele for the C677T polymorphism and C-allele for the A1298C polymorphism.

2 Hardy-Weinberg equilibrium (HWE) was calculated using ψ2-test. *p*-values <0.05 were considered not in agreement with HWE

3 Source of controls.

4 Score was calculated using Newcastle–Ottawa Quality Assessment Scale, a score <6 indicates high bias.

5 Cases and controls have Down syndrome.

### Effect of C677T polymorphism on hematological malignancies development

All models presented significant heterogeneity, analyzed using the random effects model, except for the heterozygous model in which the fixed effects model was used. The C677T polymorphism showed a decreased risk for developing cancer in only the heterozygous genetic model (OR=0.86, 95%CI=0.74-0.99, *p*=0.04, Table 2). The other models did demonstrate a decreased risk, but failed to achieve significance. All forest plots are available as supplementary material (Figures S2-   S6).

**Table 2:** Association between the MTHFR polymorphisms and developing hematological cancers in Latin Americans.

		Association ^a^			Heterogeneity ^b^		
Mutation	Genetic Model	OR	95%CI	*p*-value	Effect Model	*p*-value	I^2^
*C677T*							
	Heterozygous	0.86	0.74 – 0.99	0.04*	Fixed	0.31	12%
	Homozygous	0.97	0.67 – 1.26	0.59	Random	0.05	41%
	Dominant	0.87	0.72 – 1.05	0.14	Random	0.05	42%
	Recessive	0.90	0.62 – 1.32	0.60	Random	<0.01	65%
	Allelic	0.94	0.79 – 1.10	0.43	Random	<0.01	58%
*A1298C*							
	Heterozygous	1.04	0.82 – 1.32	0.76	Random	<0.01	55%
	Homozygous	1.69	1.11 – 2.56	0.01 *	Random	0.08	39%
	Dominant	1.19	0.97 – 1.46	0.10	Random	0.04	46%
	Recessive	1.58	1.19 – 2.08	<0.01*	Fixed	0.16	28%
	Allelic	1.21	1.00 – 1.46	0.05	Random	<0.01	59%

a Odds ratios (OR) and 95% confidence intervals (95%CI) were calculated by Revman v5.3. *p*-values <0.05 are considered significant and indicated by *.

b Heterogeneity was determined by calculated Cochran’s Q test (p-value) and the Inconsistency Index (I^2^). Significant heterogeneity was considered when the *p*-value <0.10 and I^2^>40%.

For each genetic model, the stability of the results was determined by re-calculating the pooled ORs after removal of one study. For the heterozygous genetic model, removal of either Franco 2001 (OR=0.88, 95%CI: 0.76-1.02), Metayer 2011 (OR=87, 95%CI: 0.75-1.02), Silva 2013 (ALL) (OR=0.90, 95%CI: 0.78-1.05), Silva 2013 (AML) (OR=0.87, 95%CI: 0.75-1.01), or Zanrosso 2006 (OR=0.88, 95%CI: 0.76-1.03) led to a loss of significance of pooled ORs. None of the other genetic models were sensitive to any of the publications (Figure S7).

**Figure 2:**
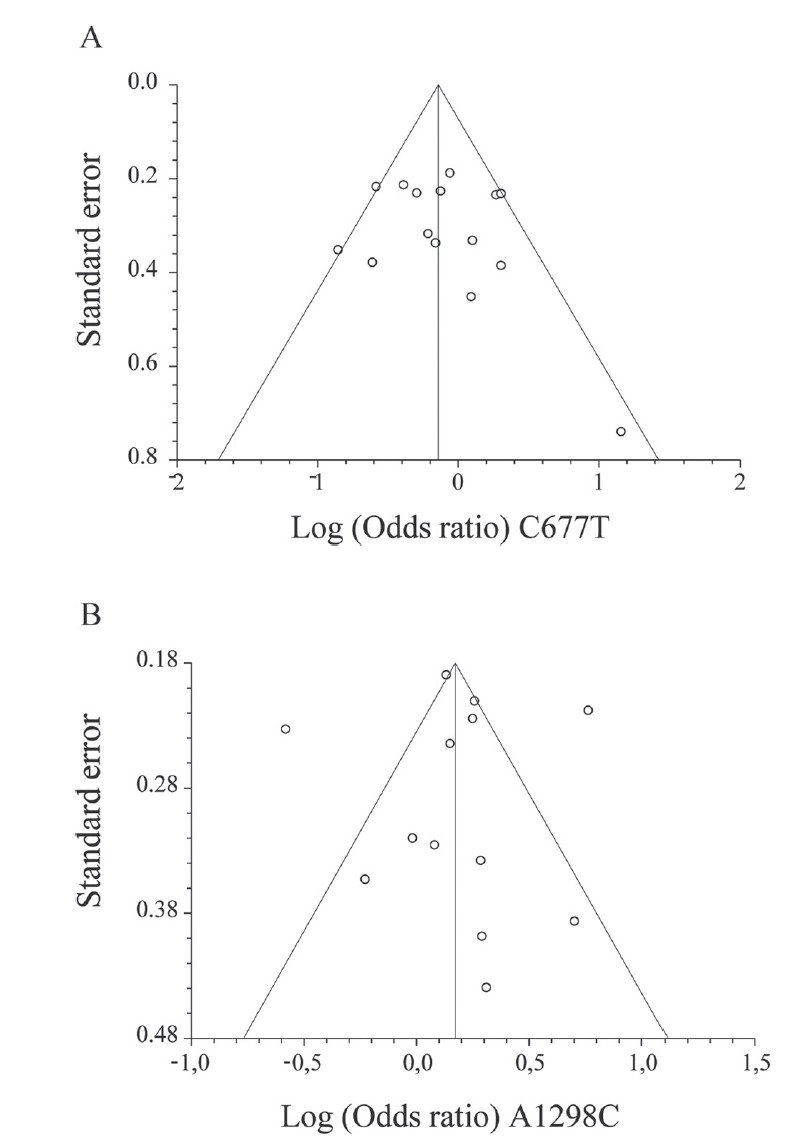
Begg’s funnel plot for publication bias test. For the MTHFR C677T (A) and A1298C (B) polymorphisms, no detrimental asymmetry was observed (dominant model). Each point represents a separate study. Similar results were determined for all other genetic models.

Publication bias was assessed by examining the funnel plot for each genetic model. Funnel plots demonstrated no significant asymmetry and the shape of the funnel plot suggested no evidence of publication bias (Figure 2A and Figure S8). Moreover, no correlation was determined by the Begg-Mazumdar’s test or bias by Egger’s Test for each model (Homozygous model: Kendall’s tau=0.668, *p*=0.99 and Egger’s Test: bias = 0.48, *p*=0.65; Heterozygous model: Kendall’s tau=0.30, p=0.14 and Egger’s Test: bias = 0.65, *p*=0.51; Dominant model: Kendall’s tau = 0.16, *p*=0.44 and Egger’s Test: bias = 0.81, *p*=0.50; Recessive model: Kendall’s tau = 0.09, *p*=0.70 and Egger’s Test: bias = 0.64, *p*=0.66; and Allelic model: Kendall’s tau = 0.10, *p*=0.63 and Egger’s Test: bias = 0.83, *p*=0.58).

### Effect of A1298C polymorphism on hematological malignancy development

All models presented with significant heterogeneity and were analyzed using the random effects model, except for the Recessive model in which the Fixed Effects model was used. The A1298C polymorphism showed an increased risk of developing hematologic malignancies for the homozygous (OR=1.69, 95%CI=1.11-2.56, *p*=0.01) and recessive (OR=1.58, 95%CI=1.19-2.08, *p*<0.01) genetic models (Table 2). Interestingly, the Allelic genetic model almost achieved significance (*p*=0.05). All forest plots are available as supplementary material (Figures S9-   S13).

When the stability of the results was examined, the homozygous and heterozygous genetic models were resistant to changes in the pooled ORs (Figure S14). However, for the dominant genetic model, removal of the HWE-inconsistence study (Lordelo *et al.*, 2012) resulted in significance (OR=1.30, 95%CI: 1.11-1.51). For the Recessive genetic model, removal of only the Silva *et al.* (2013) study resulted in loss of significance (OR=1.32, 95%CI: 0.96-1.81). The Allelic genetic model showed sensitivity to two studies (Lordelo *et al.*, 2012; OR=1.30, 95%CI: 1.11-1.52, and Zanrosso *et al.*, 2005; OR=1.25, 95%CI: 1.03-1.51).

When publication bias was assessed, no significant asymmetry was determined by examining the funnel plots (Figure 2B and Figure S15). Moreover, this was confirmed by the Begg-Mazumdar’s test and Egger’s Test (Homozygous model: Kendall’s tau =0.21, *p*=0.31 and Egger’s Test: bias = -1.29, *p*=0.18; Heterozygous model: Kendall’s tau =0.10, *p*=0.68 and Egger’s Test: bias =1.44, *p*=0.40; Dominant model: Kendall’s tau = 0.05, *p*=0.77 and Egger’s Test: bias = 0.09, *p*=0.96; Recessive model: Kendall’s tau = 0.23, *p*=0.25 and Egger’s Test: bias = -1.11, *p*=0.20; and Allelic model: Kendall’s tau = 0.10, *p*=0.68 and Egger’s Test: bias = 0.60, *p*=0.73).

### The contrary effects of the C677T and the A1298C polymorphisms in ALL

When hematologic malignancies were separated by type of cancer, neither of the polymorphisms had an effect on AML, CML, or MM (Table 3). For ALL, we excluded the Ruiz-Argüelles *et al.* (2009) study due to the high level of bias and the lack of local controls. For each model, the C677T polymorphism was associated with a significant decrease in risk of developing ALL (OR range=0.54-0.75, *p*<0.01), whereas the A1298 polymorphism was associated with an increased risk of developing ALL (OR range=1.28-2.52, *p*<0.05). This suggests that the MTHFR polymorphisms have a dual function among ALL cases.

**Table 3:** Association between the MTHFR polymorphisms and developing hematological cancers, stratified by type of cancer.

Type of cancer	n ^a^	Genetic Model	OR ^b^	95% CI ^b^	*p*-value ^b^
*C677T*					
ALL	7	Heterozygous	0.71	0.58 – 0.87	< 0.01*
	7	Homozygous	0.62	0.46 – 0.85	< 0.01*
	7	Dominant	0.68	0.56 – 0.83	< 0.01*
	7	Recessive	0.54	0.35 – 0.83	< 0.01*
	7	Allelic	0.75	0.65 – 0.87	<0.01*
AML	5	Heterozygous	0.89	0.68 – 1.18	0.43
	5	Homozygous	1.22	0.77 – 1.93	0.40
	5	Dominant	0.95	0.73 – 1.24	0.71
	5	Recessive	1.28	0.82 – 1.99	0.28
	5	Allelic	1.03	0.84 – 1.26	0.80
CML	2	Heterozygous	1.14	0.77 – 1.68	0.52
	2	Homozygous	1.15	0.30 – 4.37	0.84
	2	Dominant	1.14	0.74 – 1.77	0.55
	2	Recessive	1.13	0.34 – 3.68	0.84
	2	Allelic	1.09	0.67 – 1.75	0.73
MM	1	Heterozygous	1.28	0.79 – 2.07	0.32
	1	Homozygous	1.46	0.66 – 3.19	0.35
	1	Dominant	1.31	0.83 – 2.07	0.25
	1	Recessive	1.29	0.61 – 2.73	0.50
	1	Allelic	1.23	0.87 – 1.73	0.24
*A1298C*					
ALL	5	Heterozygous	1.28	1.01 – 1.62	0.04*
	5	Homozygous	2.52	1.40 – 4.56	< 0.01*
	5	Dominant	1.41	1.09 – 1.82	< 0.01*
	5	Recessive	2.25	1.48 – 3.41	< 0.01*
	5	Allelic	1.44	1.13 – 1.83	< 0.01*
AML	5	Heterozygous	0.99	0.42 – 1.66	0.98
	5	Homozygous	1.51	0.86 – 2.64	0.15
	5	Dominant	1.20	0.92 – 1.57	0.18
	5	Recessive	1.30	0.80 – 2.10	0.29
	5	Allelic	1.19	0.92 – 1.55	0.19
CML	2	Heterozygous	0.77	0.42 – 1.42	0.40
	2	Homozygous	0.47	0.09 – 2.42	0.37
	2	Dominant	0.75	0.39 – 1.42	0.37
	2	Recessive	0.49	0.16 – 1.52	0.21
	2	Allelic	0.78	0.48 – 1.27	0.31
MM	1	Heterozygous	1.08	0.64 – 1.83	0.77
	1	Homozygous	1.47	0.62 – 3.50	0.38
	1	Dominant	1.16	0.72 – 1.87	0.54
	1	Recessive	1.44	0.61 – 3.38	0.40
	1	Allelic	1.20	0.81 – 1.77	0.38

a Number of studies included in the analysis.

b OR and 95%CI were calculated by Revman v5.3. *p*-values<0.05 are considered significant and indicated by *.

## Discussion

This is the first meta-analysis to solely examine hematologic malignancies in Latinos. Some of the studies used here have been examined in other meta-analyses; however, the studies have been included in an overall “mixed” group, which included other non-Latino populations. For example, the two most complete meta-analyses, Zhu *et al*. and Xie *et al*., included eleven of the studies used here that were categorized as mixed, which also included studies from USA (Xie *et al.*, 2015, Zhu *et al.*, 2016)[Bibr B45] [Bibr B46]. However, in these meta-analyses, they did not classify hematologic malignancies as ALL, AML, CML, and MM. Moreover, neither study examined their “mixed” group or Latinos specifically. This is also seen in other meta-analyses that focused on hematologic malignancies. Thus, this report does clarify the risk associated with the MTHFR polymorphisms and hematologic malignancies in Latinos.

In Latinos, the MTHFR polymorphisms were not associated with CML, AML, or MM, which is consistent with other populations. For AML and CML, Qin *et al*. determined that in Caucasians and Asians, neither MTHFR polymorphism augment the risk of developing cancer (Qin *et al.*, 2014). Li *et al.* (2015a) determined that for the C677T polymorphism, there was no effect on cancer development, which is consistent with another study (Dong *et al.*, 2014), as well as our results. However, the Li *et al.* (2015a) study does demonstrate that the A1298C polymorphism was associated with an increased risk of developing CML in Asians and not Caucasians. Here, no effect was found; however, this could be due to small sample size or the fact that the Asian ethnicity has minimal influence among the majority of Latinos. Interestingly, we had only one study that focused on MM and neither polymorphism was associated with an effect. This is in agreement with Ma *et al.* (2009); however, when they only used “intermediate quality” studies, there was a significant increase in risk for MM (Ma *et al.*, 2009). The MM study used here (Lima), was determined to be of intermediate quality by our scoring system, thus we posit that more studies on the Latino population focusing on MM could demonstrate an association between the C677T polymorphism and MM (Lima *et al.*, 2008).

Interestingly, we found a dual effect of the MTHFR polymorphisms for ALL. Here, the A1298C polymorphism was shown to increase the risk of developing ALL by 1.3- to 2.5-fold, whereas for the C677T polymorphism, the ORs ranged between 0.70-0.90. Other studies typically do not show a similar result. For example, Li *et al*. demonstrated no effect for either polymorphism (Li *et al.*, 2015b). However, Jiang *et al.* (2013) showed that for Caucasians, the C677T polymorphism decreased the risk, which is not shared with their Asians and Others groups. Interestingly, Zhang *et al.* (2017) demonstrated no affect for the C677T polymorphisms in their “mixed” group; however, a significant decrease in risk for the Asians and Caucasians was observed (Zhang *et al.*, 2017). Moreover, Xie *et al.* (2015) demonstrated a significant association between the C677T polymorphism in ALL in adults and children for Caucasians and Asians, respectively. However, they did not examine their “mixed” group, and the analyses that included Latinos were a combination of CML, AML, and ALL, without indicating their proportions. This could mask the effect of ALL, as seen with our data. For ALL, a majority of the studies focused on children, with only 1 study on adults. For the C677T, Latino children were shown to have an increased risk, which was not shared with the adult study (no risk). With few studies focusing on adults, we can only assume that the C677T polymorphism has no effect, and this is in accordance with Li *et al.* (2015b). For A1298C, all studies focused on childhood onset. Most meta-analyses have shown no effect in developing ALL (Yan *et al.*, 2012, Zhu *et al.*, 2016); however, here we clearly show that the C allele is associated with an increased risk.

A key factor that must be considered is the genetic diversity of Latin America and the Caribbean populations. In Mexico, the genetic composition derives from Native Americans, Europeans, and Africans, which significantly fluctuate from region to region (Moreno-Estrada *et al.*, 2013). This phenomenon is also seen among different regions of Brazil (Pena *et al.*, 2009, Ramos *et al.*, 2016). These differences lead to various development rates and pathologies of similar diseases. For example, it was shown that the level of Native ancestry has a significant impact on lung function among the Mexican population (Moreno-Estrada *et al.*, 2013). However, due to the few studies available, determining the effect that genetic composition has on hematologic malignancies remains elusive. Thus, more studies are required with a focus on the genetic make-up of the subjects.

In Latin America, the consumption of folic acid and other parts of the folate pathway (Vitamin B12 and B6) is low compared to other regions of the world (Brito *et al.*, 2015). Under low folate consumption, the folate pathway cannot convert homocysteine to methionine, abrogating DNA methylation (Crider *et al.*, 2012). Interestingly, here we showed that for the C677T polymorphism, the T allele is associated with a decreased risk of developing ALL. This “protective factor” has been determined with other cancers (Zhao *et al.*, 2013; Guo *et al.*, 2015). The proposed mechanism for this protective effect has not been fully elucidated; however, it is believed that the severe loss of enzymatic activity leads to a switch from DNA methylation to promote dTMP synthesis from 5,10-methylenetetrahydrofolate (Blount *et al.*, 1997). The less active A1298C polymorphism still allows DNA methylation, promoting oncogene expression and decreasing tumor suppressor gene expression. In support of this, it was shown that the T-allele allows a faster dissociation of central stabilizing cofactors, decreasing the activity of MTHFR (Tang *et al.*, 2014).

One concern with our results is the coverage of Latin America. Here, three countries/regions were examined (Brazil, Mexico, and Latin Americans living in the USA). We initial hoped that including alternative databases — LILACS, BIBLAT, LATININDEX, PERIODICA, and REDALYC to name a few — would increase the coverage; however, there remained a significant underrepresentation of Latin America. Moreover, the ability to search and export the citations was problematic. This highlights the problems for research and dissemination of information that occurs among Latin American countries and suggests that studies that were presented at national conferences or regional scientific meetings could have been missed.

Our study has a few limitations. First, only three countries are represented in this meta-analysis, which suggests that parts of the Latin American community are underrepresented. Second, we calculated the crude ORs from genotype distributions and they are unadjusted estimations. Adjusting the OR for an age category (adults versus children) could influence the OR, possibly affecting the significances of our results. However, we were focusing on risk and not the age of onset. Moreover, we did not adjust the ORs for the distribution of males and females. Lastly, dietary folic acid consumption was shown to affect the risk associated with cancer development. Here, minimal studies stratified by diet and we were unable to correct for this.

## Conclusion

Here, we report the risk of hematologic malignancies associated with the two main polymorphisms of the MTHFR gene in Latin Americans. There was a significant association with ALL and not with CML, AML, or MM. The A1298C polymorphism was associated with an increased risk of developing ALL, whereas the C677T polymorphism was associated with a decreased risk, being a protective factor.

## Supplementary material

The following online material is available for this article:

Figure S1 Risk of bias by domain from all case-control studies

Figure S2 C677T Heterozygous Model

Figure S3 C677T Homozygous Model

Figure S4 C677T Dominant Model

Figure S5 C677T Recessive Model

Figure S6 C677T Allelic Model

Figure S7 Sensitivity Analysis for C677T Models

Figure S8 Publication Bias for C677T for Models

Figure S9 A1298C Heterozygous Model

Figure S10 A1298C Homozygous Model

Figure S11 A1298C Dominant Model

Figure S12 A1298C Recessive Model

Figure S13 A1298C Allelic Model

Figure S14 Sensitivity Analysis for A1298C Models

Figure S15 Publication Bias for A1298C for Models

Table S1 Prima Checklist

Table S2 Search used for MTHFR polymorphisms and cancers that focus on the Latino population

Table S3 Assessment study quality based on the Newcastle-Ottawa scale.
