# Supplementary material for: Differential effects of the methylenetetrahydrofolate reductase polymorphisms (C677T and A1298C) on hematological malignancies among Latinos: a meta-analysis
Source: Genet Mol Biol. 2019 Nov 14;42(3):549–59. doi: 10.1590/1678-4685-GMB-2018-0161 (PMC6905449; doi:10.1590/1678-4685-GMB-2018-0161)
Supplement: Supplementary file 1 [file 1415-4757-GMB-42-3-2018-0161-suppl16.pdf]

Supplementary Material to "Differential effects of the methylenetetrahydrofolate reductase polymorphisms (C677T and A1298C) on hematological malignancies among Latinos: a meta-analysis"

Table S1 - Search used for MTHFR polymorphisms and cancers on the Latino population.

(mexico OR mexican OR mexic* OR hispanic OR hispan* OR Latin* OR latin OR latino OR argentina OR argentinian OR argentinean OR argentina OR argentin* OR bolivia OR bolivian OR bolivia* OR brazil OR brazilian OR brazil* OR chile OR chilean OR chile* OR colombia OR colombian OR colombi* OR costa OR costarricense OR costarriquense OR cuba OR cuban OR cuban* OR ecuador OR ecuadorian OR ecuadorian OR ecuad* OR ecuat* OR salvador OR salvadoran OR salvadoreno OR salvador* OR guatemala OR guatemalan OR guatemalteco OR guatemal* OR haiti OR haitian OR haitian OR hait* OR honduras OR honduran OR honduran OR hondur* OR nicaragua OR nicaraguan OR nicaraguense OR nicarag* OR panama OR panameno OR panamanian OR panam* OR paraguay OR paraguayan OR paraguay OR Paragua* OR peru OR peruvian OR peruana OR peru* OR dominicana OR dominicanus OR dominican OR dominic* OR uruguay OR uruguay OR uruguayan OR Urugu* OR venezuela OR venezolano OR venezuelan OR venez* or anguila or anguilla or anguilense or Anguillan or Antigua or antiguano or antiguanobarbudense or Antigu* or aruba or arubano or arubian or arub* or Bahamas or bahameno or baham* or Barbados or barbadense or barbad* or belize or belice or beliceno or belicense or beliz* or belic* or Granada or granadino or Granad* or Guyana or guyanes or cayman or islands or caimans or caimanes or caiman* or Jamaica or jamaicano or jamaiquino or jamaic or jamai* or Trinidad or Tobago or trinitense or trinitario or trini* or vicente or sanvicentino or sanvicentin or sanvincen* or lucia or santalucense or suriname or surinamesa or Surinam* or falkland or Malvinas or malvi* or malvinense) AND (MTHFR or methylenetetrahydrofolate or metilentetrahidrofolato) AND (deletion or delet* or polymorphism or polymor* or SNP or addition or duplication or duplicat* or insert or insert*) AND (cancer or carcinom* or carcinog* or Adenoma or Adenoc* or tumor or tumour or neoplasia or neoplasm* or metaplasia or metaplas*)
