# Supplementary material for: Differential effects of the methylenetetrahydrofolate reductase polymorphisms (C677T and A1298C) on hematological malignancies among Latinos: a meta-analysis
Source: Genet Mol Biol. 2019 Nov 14;42(3):549–59. doi: 10.1590/1678-4685-GMB-2018-0161 (PMC6905449; doi:10.1590/1678-4685-GMB-2018-0161)
Supplement: Supplementary file 3 [file 1415-4757-GMB-42-3-2018-0161-suppl18.pdf]

Supplementary Material to "Differential effects of the methylenetetrahydrofolate reductase polymorphisms (C677T and A1298C) on hematological malignancies among Latinos: a meta-analysis"

Table S3 - Assessment of study quality based on the Newcastle-Ottawa scale.

	Author (year)	Is the case definition adequate?	Representativeness of the cases	Selection of controls	Definition of controls	Comparability of cases and controls on the basis of the design or analysis	Ascertainment of exposure	Same method of ascertainment for cases and controls	Non-response rate	Total score
1	Amorim, 2008	★	★	★[M]	★	★☆	★	★	☆	**7**
2	Barbosa, 2008	★	★	★[R]	☆	★☆	★	★	☆	**6**
3	da Costa Ramos, 2006	★	★	★[M]	★	★☆	★	★	☆	**7**
4	Franco, 2001	★	★	★	★	★★	★	★	☆	**8**
5	Gallegos-Arreola, 2009	★	★	★	☆	★☆	★	★	☆	**6**
6	Gutierrez-Alvarez, 2016	★	★	★[M]	★	★★	★	★	☆	**8**
7	Lima, 2008	★	★	★	☆	★☆	★	☆	☆	**6**
8	Lordelo, 2012	★	★	★	☆	★★	★	★	☆	**7**
9	Metayer, 2011	★	★	★[M]	★	★★	★	★	☆	**8**
10	Ruiz-Arguelles, 2007	★	★	★	☆	★☆	☆	☆	☆	**3**
11	Silva, 2013	★	★	★[R]	★	★☆	★	★	☆	**7**
12	Zanrosso, 2005	★	★	★[R]	★	★★	★	★	☆	**8**
13	Zanrosso, 2006	★	★	★[R]	★	★☆	☆	★	☆	**6**

[R] Indicates Regional Hospital.
[M] Indicates Multicenter Hospital-based study.
