# Supplementary material for: Differential effects of the methylenetetrahydrofolate reductase polymorphisms (C677T and A1298C) on hematological malignancies among Latinos: a meta-analysis
Source: Genet Mol Biol. 2019 Nov 14;42(3):549–59. doi: 10.1590/1678-4685-GMB-2018-0161 (PMC6905449; doi:10.1590/1678-4685-GMB-2018-0161)
Supplement: Supplementary file 5 [file 1415-4757-GMB-42-3-2018-0161-suppl2.pdf]

Supplementary Material to "Differential effects of the methylenetetrahydrofolate reductase polymorphisms (C677T and A1298C) on hematological malignancies among Latinos: a meta-analysis"

Study or Subgroup	Experimental Events	Experimental Total	Control Events	Control Total	Weight	Odds Ratio M-H, Fixed, 95% CI
**3.1.1 ALL**						
17 Franco 2001 ALL	28	64	36	58	5.3%	0.48 [0.23, 0.98]
24 Gutierrez-Alvarez 2016 ALL	36	58	72	114	4.6%	0.95 [0.50, 1.83]
35 Metayer 2011 ALL	72	134	91	150	10.0%	0.75 [0.47, 1.21]
43 Ruiz-Arguelles 2007 ALL	10	12	384	539		Not estimable
44 Silva 2013 ALL	53	135	108	203	13.1%	0.57 [0.37, 0.88]
50 Zanrosso 2006 ALL	56	152	82	178	12.0%	0.68 [0.44, 1.06]
54 Gallegos- Arreola 2008 ALL	78	142	79	138	9.1%	0.91 [0.57, 1.46]
**Subtotal (95% CI)**		**685**		**841**	**54.1%**	**0.71 [0.58, 0.87]**
Total events	323		468			
Heterogeneity: Chi² = 4.09, df = 5 (P = 0.54); I² = 0%						
Test for overall effect: Z = 3.28 (P = 0.001)						
**3.1.2 AML**						
01 Amorim 2008 AML	20	44	25	60	2.9%	1.17 [0.53, 2.56]
02 Barbosa 2008 AML	8	25	29	94	2.1%	1.05 [0.41, 2.72]
10 da Costa Ramos 2006 AML	67	160	128	284	13.5%	0.88 [0.59, 1.30]
45 Silva 2013 AML	12	31	108	203	4.4%	0.56 [0.26, 1.20]
49 Zanrosso 2005 AML	17	38	95	218	3.9%	1.05 [0.52, 2.10]
**Subtotal (95% CI)**		**298**		**859**	**26.7%**	**0.89 [0.68, 1.18]**
Total events	124		385			
Heterogeneity: Chi² = 2.22, df = 4 (P = 0.69); I² = 0%						
Test for overall effect: Z = 0.79 (P = 0.43)						
**3.1.3 CML**						
03 Barbosa 2008 CML	19	65	29	94	4.2%	0.93 [0.46, 1.85]
33 Lordelo 2012 CML	47	93	114	254	7.6%	1.25 [0.78, 2.02]
**Subtotal (95% CI)**		**158**		**348**	**11.8%**	**1.14 [0.77, 1.68]**
Total events	66		143			
Heterogeneity: Chi² = 0.50, df = 1 (P = 0.48); I² = 0%						
Test for overall effect: Z = 0.65 (P = 0.52)						
**3.1.4 MM**						
29 Lima 2008 MM	57	109	79	171	7.4%	1.28 [0.79, 2.07]
**Subtotal (95% CI)**		**109**		**171**	**7.4%**	**1.28 [0.79, 2.07]**
Total events	57		79			
Heterogeneity: Not applicable						
Test for overall effect: Z = 0.99 (P = 0.32)						
**Total (95% CI)**		**1250**		**2219**	**100.0%**	**0.85 [0.74, 0.98]**
Total events	570		1075			
Heterogeneity: Chi² = 14.79, df = 13 (P = 0.32); I² = 12%						
Test for overall effect: Z = 2.19 (P = 0.03)						
Test for subgroup differences: Chi² = 8.00, df = 3 (P = 0.05), I² = 62.5%						

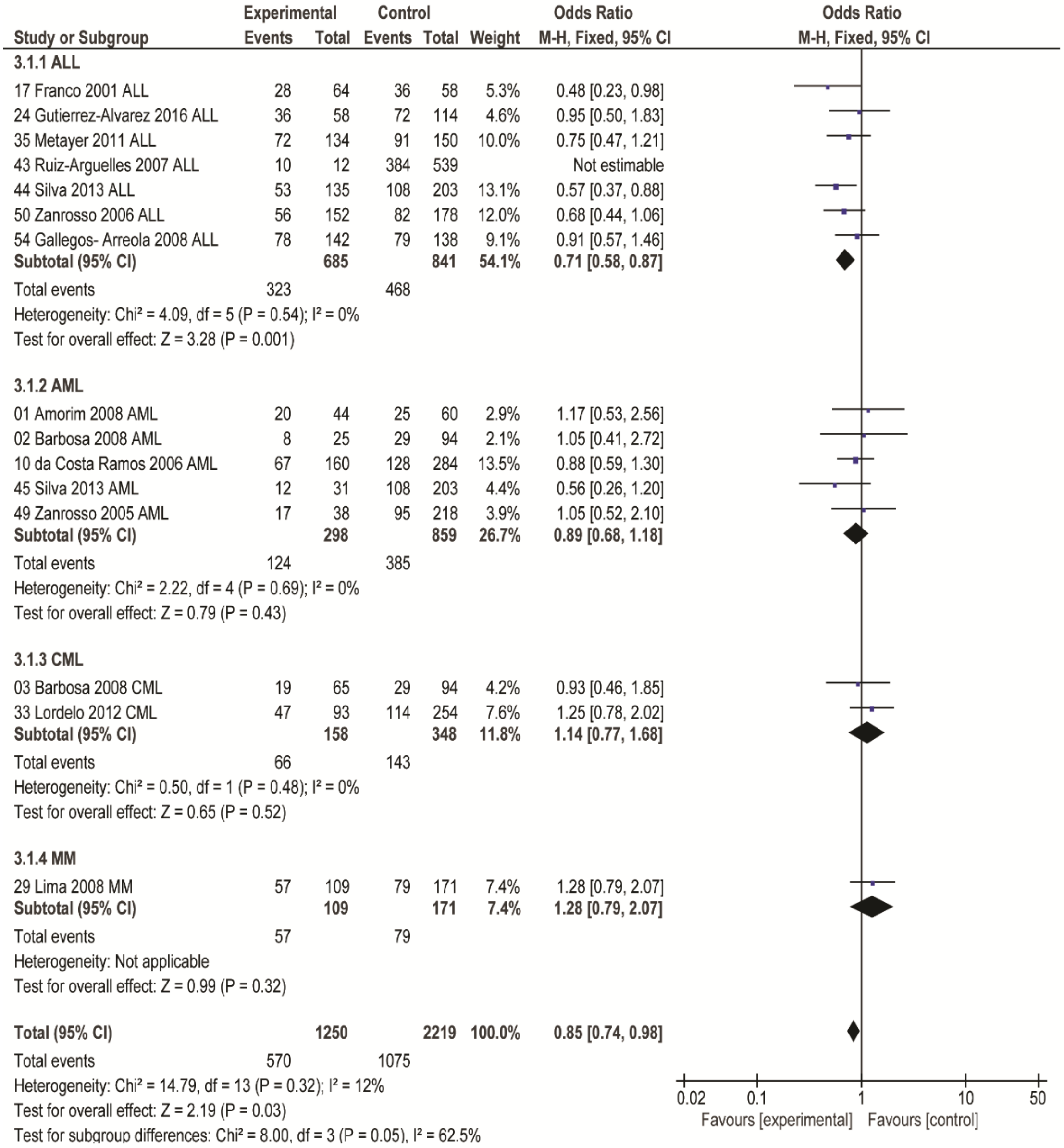

Figure S2 - C677T Heterozygous Model.
