# Supplementary material for: Differential effects of the methylenetetrahydrofolate reductase polymorphisms (C677T and A1298C) on hematological malignancies among Latinos: a meta-analysis
Source: Genet Mol Biol. 2019 Nov 14;42(3):549–59. doi: 10.1590/1678-4685-GMB-2018-0161 (PMC6905449; doi:10.1590/1678-4685-GMB-2018-0161)
Supplement: Supplementary file 6 [file 1415-4757-GMB-42-3-2018-0161-suppl3.pdf]

Supplementary Material to "Differential effects of the methylenetetrahydrofolate reductase polymorphisms (C677T and A1298C) on hematological malignancies among Latinos: a meta-analysis"

Study or Subgroup	Experimental Events	Experimental Total	Control Events	Control Total	Weight	Odds Ratio M-H, Random, 95% CI
**3.2.1 ALL**						
17 Franco 2001 ALL	6	42	13	35	5.4%	0.28 [0.09, 0.85]
24 Gutierrez-Alvarez 2016 ALL	12	34	38	80	8.3%	0.60 [0.26, 1.38]
35 Metayer 2011 ALL	20	82	27	86	10.6%	0.70 [0.36, 1.39]
43 Ruiz-Arguelles 2007 ALL	16	18	251	406		Not estimable
44 Silva 2013 ALL	9	91	21	116	8.2%	0.50 [0.22, 1.14]
50 Zanrosso 2006 ALL	13	109	20	116	9.3%	0.65 [0.31, 1.38]
54 Gallegos- Arreola 2008 ALL	28	92	32	91	11.8%	0.81 [0.43, 1.50]
**Subtotal (95% CI)**		**450**		**524**	**53.6%**	**0.62 [0.46, 0.85]**
Total events	88		151			
Heterogeneity: Tau² = 0.00; Chi² = 3.08, df = 5 (P = 0.69); I² = 0%						
Test for overall effect: Z = 2.97 (P = 0.003)						
**3.2.2 AML**						
01 Amorim 2008 AML	5	29	2	37	2.6%	3.65 [0.65, 20.36]
02 Barbosa 2008 AML	2	19	6	71	2.7%	1.27 [0.24, 6.89]
10 da Costa Ramos 2006 AML	22	115	31	187	12.1%	1.19 [0.65, 2.18]
45 Silva 2013 AML	2	21	21	116	3.1%	0.48 [0.10, 2.20]
49 Zanrosso 2005 AML	5	26	22	145	5.7%	1.33 [0.45, 3.90]
**Subtotal (95% CI)**		**210**		**556**	**26.1%**	**1.22 [0.77, 1.93]**
Total events	36		82			
Heterogeneity: Tau² = 0.00; Chi² = 3.04, df = 4 (P = 0.55); I² = 0%						
Test for overall effect: Z = 0.84 (P = 0.40)						
**3.2.3 CML**						
03 Barbosa 2008 CML	2	48	6	71	2.8%	0.47 [0.09, 2.44]
33 Lordelo 2012 CML	12	58	19	159	8.7%	1.92 [0.87, 4.26]
**Subtotal (95% CI)**		**106**		**230**	**11.5%**	**1.15 [0.30, 4.37]**
Total events	14		25			
Heterogeneity: Tau² = 0.56; Chi² = 2.30, df = 1 (P = 0.13); I² = 57%						
Test for overall effect: Z = 0.21 (P = 0.84)						
**3.2.4 MM**						
29 Lima 2008 MM	14	66	17	109	8.9%	1.46 [0.66, 3.19]
**Subtotal (95% CI)**		**66**		**109**	**8.9%**	**1.46 [0.66, 3.19]**
Total events	14		17			
Heterogeneity: Not applicable						
Test for overall effect: Z = 0.94 (P = 0.35)						
**Total (95% CI)**		**832**		**1419**	**100.0%**	**0.86 [0.65, 1.15]**
Total events	152		275			
Heterogeneity: Tau² = 0.09; Chi² = 18.61, df = 13 (P = 0.14); I² = 30%						
Test for overall effect: Z = 1.00 (P = 0.32)						
Test for subgroup differences: Chi² = 8.13, df = 3 (P = 0.04), I² = 63.1%						

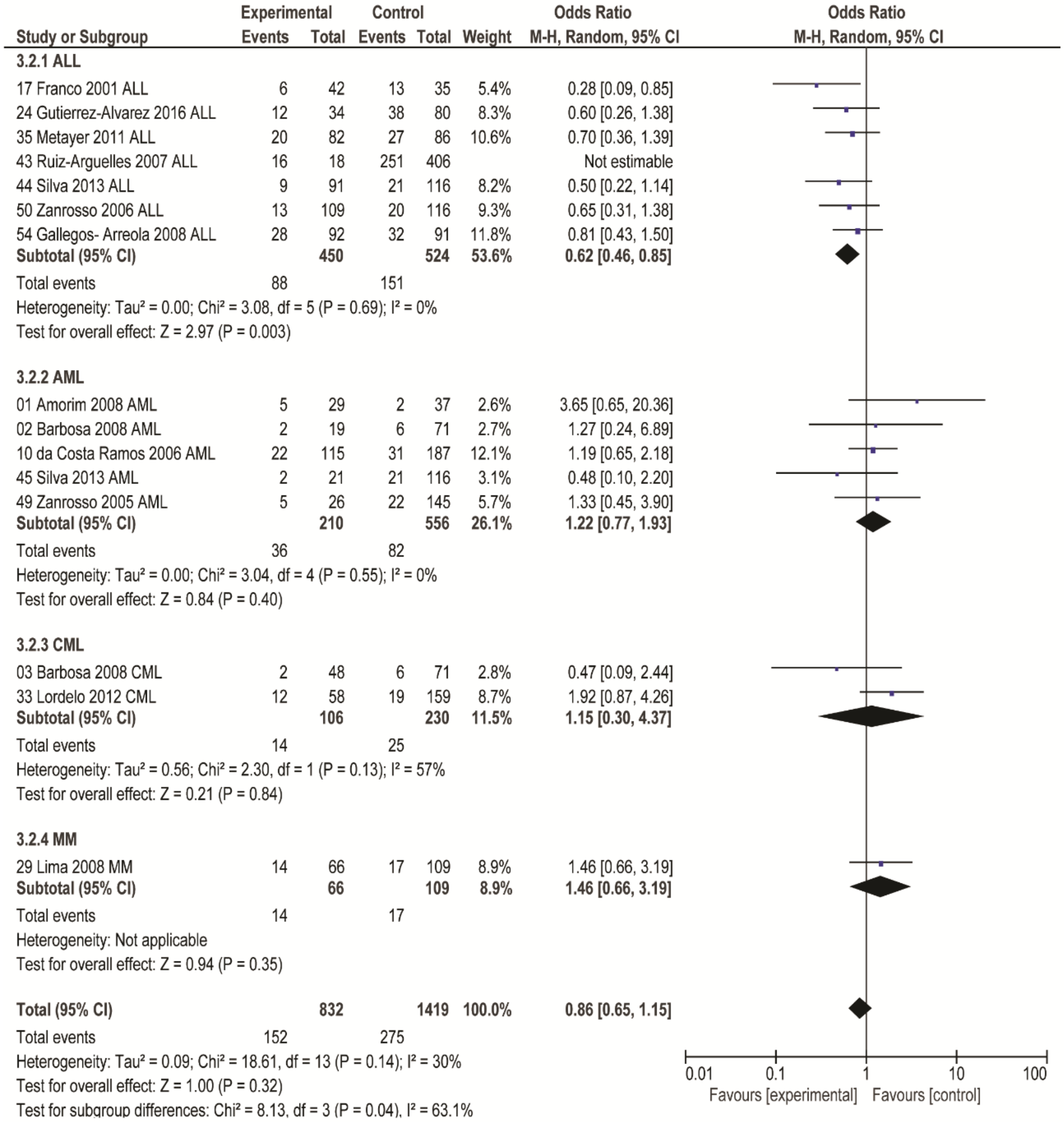

Figure S3 - C677T Homozygous Model.
