# Supplementary material for: Differential effects of the methylenetetrahydrofolate reductase polymorphisms (C677T and A1298C) on hematological malignancies among Latinos: a meta-analysis
Source: Genet Mol Biol. 2019 Nov 14;42(3):549–59. doi: 10.1590/1678-4685-GMB-2018-0161 (PMC6905449; doi:10.1590/1678-4685-GMB-2018-0161)
Supplement: Supplementary file 7 [file 1415-4757-GMB-42-3-2018-0161-suppl4.pdf]

Supplementary Material to "Differential effects of the methylenetetrahydrofolate reductase polymorphisms (C677T and A1298C) on hematological malignancies among Latinos: a meta-analysis"

Study or Subgroup	Experimental Events	Experimental Total	Control Events	Control Total	Weight	Odds Ratio M-H, Random, 95% CI
**3.3.1 ALL**						
17 Franco 2001 ALL	34	70	49	71	5.1%	0.42 [0.21, 0.84]
24 Gutierrez-Alvarez 2016 ALL	48	70	111	152	5.9%	0.81 [0.43, 1.50]
35 Metayer 2011 ALL	92	154	118	177	8.9%	0.74 [0.47, 1.16]
43 Ruiz-Arguelles 2007 ALL	26	28	635	790		Not estimable
44 Silva 2013 ALL	62	144	129	224	9.5%	0.56 [0.36, 0.85]
50 Zanrosso 2006 ALL	69	165	102	198	9.6%	0.68 [0.45, 1.03]
54 Gallegos- Arreola 2008 ALL	106	170	111	170	9.0%	0.88 [0.57, 1.37]
**Subtotal (95% CI)**		**773**		**992**	**48.1%**	**0.68 [0.56, 0.83]**
Total events	411		620			
Heterogeneity: Tau² = 0.00; Chi² = 4.41, df = 5 (P = 0.49); I² = 0%						
Test for overall effect: Z = 3.85 (P = 0.0001)						
**3.3.2 AML**						
01 Amorim 2008 AML	25	49	27	62	4.4%	1.35 [0.64, 2.86]
02 Barbosa 2008 AML	10	27	35	100	3.4%	1.09 [0.45, 2.64]
10 da Costa Ramos 2006 AML	89	182	159	315	11.0%	0.94 [0.65, 1.35]
45 Silva 2013 AML	14	33	129	224	4.6%	0.54 [0.26, 1.14]
49 Zanrosso 2005 AML	22	43	117	240	5.5%	1.10 [0.58, 2.11]
**Subtotal (95% CI)**		**334**		**941**	**29.0%**	**0.95 [0.73, 1.24]**
Total events	160		467			
Heterogeneity: Tau² = 0.00; Chi² = 3.34, df = 4 (P = 0.50); I² = 0%						
Test for overall effect: Z = 0.37 (P = 0.71)						
**3.3.3 CML**						
03 Barbosa 2008 CML	21	67	35	100	5.4%	0.85 [0.44, 1.64]
33 Lordelo 2012 CML	59	105	133	273	8.8%	1.35 [0.86, 2.12]
**Subtotal (95% CI)**		**172**		**373**	**14.2%**	**1.14 [0.74, 1.77]**
Total events	80		168			
Heterogeneity: Tau² = 0.02; Chi² = 1.30, df = 1 (P = 0.25); I² = 23%						
Test for overall effect: Z = 0.59 (P = 0.55)						
**3.3.4 MM**						
29 Lima 2008 MM	71	123	96	188	8.7%	1.31 [0.83, 2.07]
**Subtotal (95% CI)**		**123**		**188**	**8.7%**	**1.31 [0.83, 2.07]**
Total events	71		96			
Heterogeneity: Not applicable						
Test for overall effect: Z = 1.15 (P = 0.25)						
**Total (95% CI)**		**1402**		**2494**	**100.0%**	**0.85 [0.71, 1.02]**
Total events	722		1351			
Heterogeneity: Tau² = 0.04; Chi² = 20.83, df = 13 (P = 0.08); I² = 38%						
Test for overall effect: Z = 1.74 (P = 0.08)						
Test for subgroup differences: Chi² = 10.78, df = 3 (P = 0.01), I² = 72.2%						

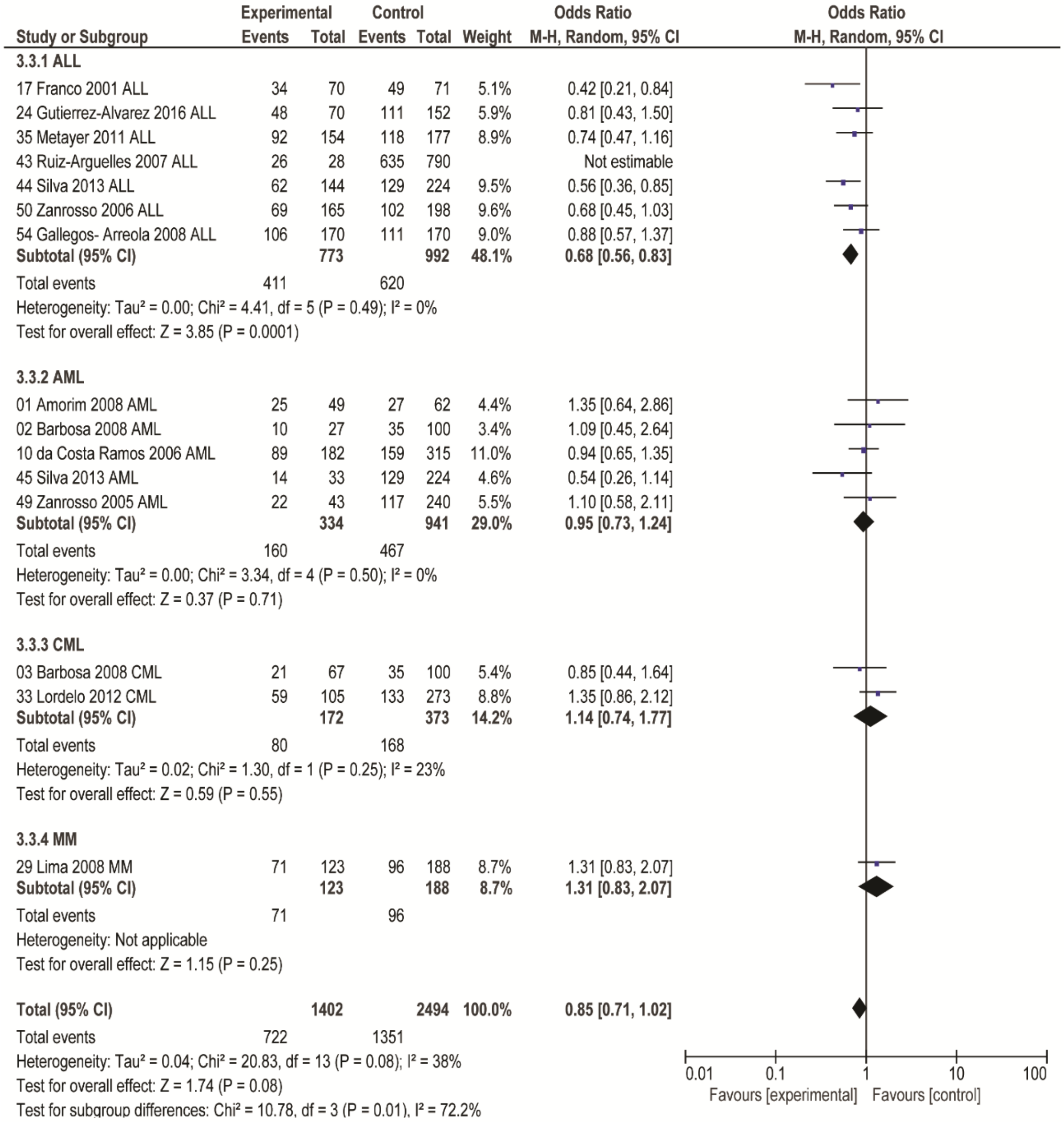

Figure S4 - C677T Dominant Model.
