# Supplementary material for: Differential effects of the methylenetetrahydrofolate reductase polymorphisms (C677T and A1298C) on hematological malignancies among Latinos: a meta-analysis
Source: Genet Mol Biol. 2019 Nov 14;42(3):549–59. doi: 10.1590/1678-4685-GMB-2018-0161 (PMC6905449; doi:10.1590/1678-4685-GMB-2018-0161)
Supplement: Supplementary file 8 [file 1415-4757-GMB-42-3-2018-0161-suppl5.pdf]

Supplementary Material to "Differential effects of the methylenetetrahydrofolate reductase polymorphisms (C677T and A1298C) on hematological malignancies among Latinos: a meta-analysis"

Study or Subgroup	Experimental Events	Experimental Total	Control Events	Control Total	Weight	Odds Ratio M-H, Random, 95% CI
**3.4.1 ALL**						
17 Franco 2001 ALL	6	70	13	71	6.4%	0.42 [0.15, 1.17]
24 Gutierrez-Alvarez 2016 ALL	12	70	38	152	8.9%	0.62 [0.30, 1.28]
35 Metayer 2011 ALL	20	154	27	177	9.8%	0.83 [0.44, 1.55]
43 Ruiz-Arguelles 2007 ALL	16	28	251	790		Not estimable
44 Silva 2013 ALL	9	144	21	224	8.1%	0.64 [0.29, 1.45]
50 Zanrosso 2006 ALL	13	165	20	198	8.8%	0.76 [0.37, 1.58]
54 Gallegos- Arreola 2008 ALL	28	170	32	70	9.8%	0.23 [0.13, 0.44]
**Subtotal (95% CI)**		**773**		**892**	**51.8%**	**0.54 [0.35, 0.83]**
Total events	88		151			
Heterogeneity: $Tau^2 = 0.14$; $Chi^2 = 10.19$, df = 5 (P = 0.07); $I^2 = 51\%$						
Test for overall effect: Z = 2.81 (P = 0.005)						
**3.4.2 AML**						
01 Amorim 2008 AML	5	49	2	62	3.4%	3.41 [0.63, 18.39]
02 Barbosa 2008 AML	2	27	6	100	3.5%	1.25 [0.24, 6.59]
10 da Costa Ramos 2006 AML	22	182	31	315	10.2%	1.26 [0.71, 2.25]
45 Silva 2013 AML	2	33	21	224	4.0%	0.62 [0.14, 2.79]
49 Zanrosso 2005 AML	5	43	22	240	6.4%	1.30 [0.47, 3.65]
**Subtotal (95% CI)**		**334**		**941**	**27.5%**	**1.28 [0.82, 1.99]**
Total events	36		82			
Heterogeneity: $Tau^2 = 0.00$; $Chi^2 = 2.19$, df = 4 (P = 0.70); $I^2 = 0\%$						
Test for overall effect: Z = 1.08 (P = 0.28)						
**3.4.3 CML**						
03 Barbosa 2008 CML	2	67	6	100	3.5%	0.48 [0.09, 2.46]
33 Lordelo 2012 CML	12	105	19	273	8.5%	1.72 [0.81, 3.69]
**Subtotal (95% CI)**		**172**		**373**	**12.1%**	**1.13 [0.34, 3.68]**
Total events	14		25			
Heterogeneity: $Tau^2 = 0.40$; $Chi^2 = 1.94$, df = 1 (P = 0.16); $I^2 = 49\%$						
Test for overall effect: Z = 0.20 (P = 0.84)						
**3.4.4 MM**						
29 Lima 2008 MM	14	123	17	188	8.6%	1.29 [0.61, 2.73]
**Subtotal (95% CI)**		**123**		**188**	**8.6%**	**1.29 [0.61, 2.73]**
Total events	14		17			
Heterogeneity: Not applicable						
Test for overall effect: Z = 0.67 (P = 0.50)						
**Total (95% CI)**		**1402**		**2394**	**100.0%**	**0.81 [0.57, 1.16]**
Total events	152		275			
Heterogeneity: $Tau^2 = 0.24$; $Chi^2 = 29.66$, df = 13 (P = 0.005); $I^2 = 56\%$						
Test for overall effect: Z = 1.13 (P = 0.26)						
Test for subgroup differences: $Chi^2 = 8.84$, df = 3 (P = 0.03), $I^2 = 66.1\%$						

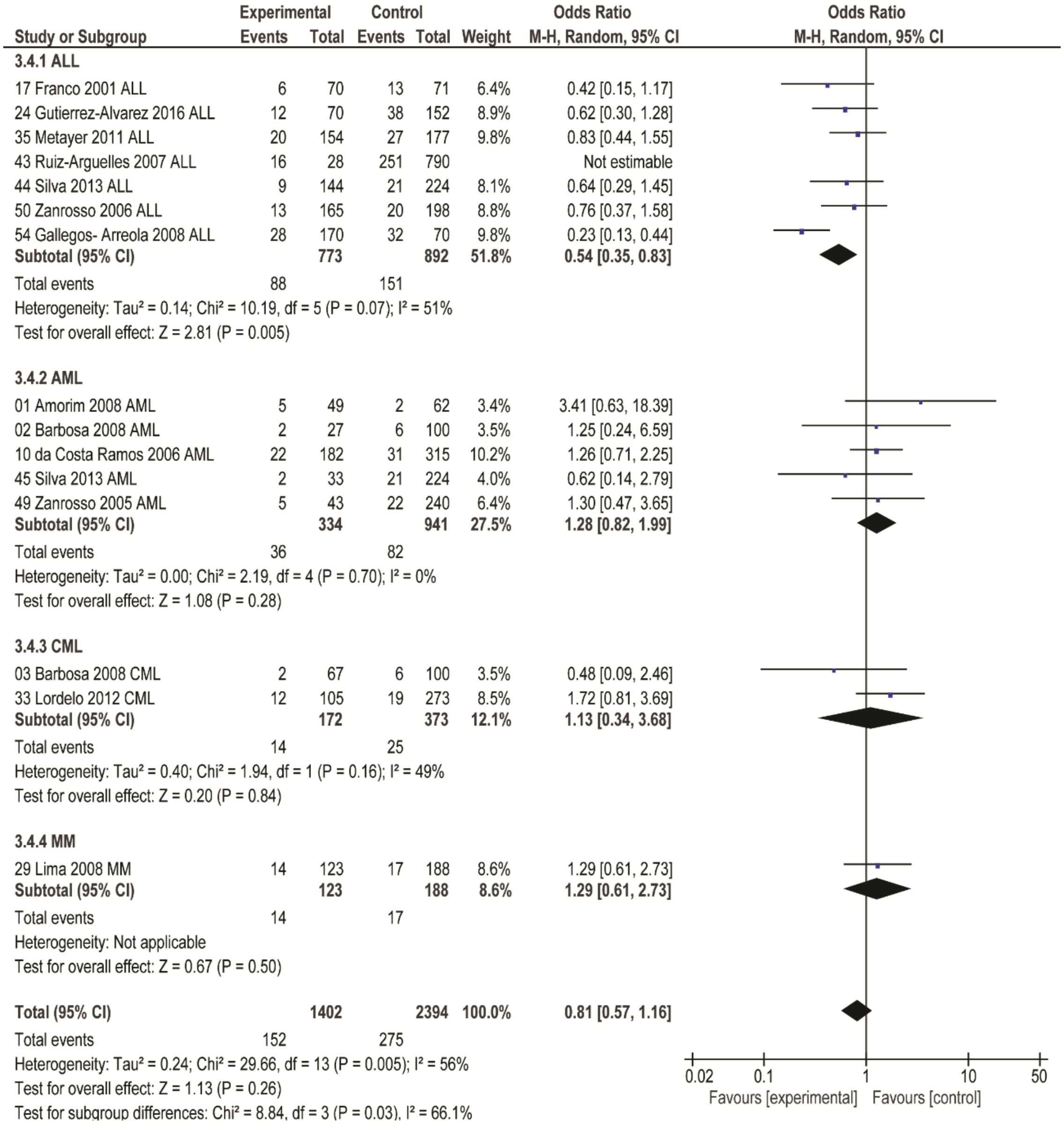

Figure S5 - C677T Recessive Model.
