# Supplementary material for: Differential effects of the methylenetetrahydrofolate reductase polymorphisms (C677T and A1298C) on hematological malignancies among Latinos: a meta-analysis
Source: Genet Mol Biol. 2019 Nov 14;42(3):549–59. doi: 10.1590/1678-4685-GMB-2018-0161 (PMC6905449; doi:10.1590/1678-4685-GMB-2018-0161)
Supplement: Supplementary file 13 [file 1415-4757-GMB-42-3-2018-0161-suppl10.pdf]

Supplementary Material to "Differential effects of the methylenetetrahydrofolate reductase polymorphisms (C677T and A1298C) on hematological malignancies among Latinos: a meta-analysis"

Study or Subgroup	Experimental Events	Experimental Total	Control Events	Control Total	Weight	Odds Ratio M-H, Random, 95% CI
**3.2.1 ALL**						
17 Franco 2001 ALL	5	41	2	43	4.7%	2.85 [0.52, 15.58]
24 Gutierrez-Alvarez 2016 ALL	6	56	2	110	5.0%	6.48 [1.26, 33.24]
35 Metayer 2011 ALL	8	94	6	116	8.8%	1.71 [0.57, 5.10]
44 Silva 2013 ALL	28	83	19	166	14.3%	3.94 [2.04, 7.62]
50 Zanrosso 2006 ALL	11	94	12	123	11.3%	1.23 [0.52, 2.91]
**Subtotal (95% CI)**		**368**		**558**	**44.1%**	**2.52 [1.40, 4.56]**
Total events	58		41			
Heterogeneity: Tau² = 0.16; Chi² = 6.20, df = 4 (P = 0.19); I² = 35%						
Test for overall effect: Z = 3.06 (P = 0.002)						
**3.2.2 AML**						
01 Amorim 2008 AML	6	36	4	44	6.7%	2.00 [0.52, 7.72]
02 Barbosa 2008 AML	1	16	5	68	3.0%	0.84 [0.09, 7.73]
10 da Costa Ramos 2006 AML	16	120	21	211	13.8%	1.39 [0.70, 2.78]
45 Silva 2013 AML	5	18	19	166	8.4%	2.98 [0.95, 9.27]
49 Zanrosso 2005 AML	1	29	18	169	3.5%	0.30 [0.04, 2.34]
**Subtotal (95% CI)**		**219**		**658**	**35.3%**	**1.51 [0.86, 2.64]**
Total events	29		67			
Heterogeneity: Tau² = 0.04; Chi² = 4.38, df = 4 (P = 0.36); I² = 9%						
Test for overall effect: Z = 1.44 (P = 0.15)						
**3.2.3 CML**						
03 Barbosa 2008 CML	3	44	5	68	5.8%	0.92 [0.21, 4.07]
33 Lordelo 2012 CML	1	62	11	130	3.4%	0.18 [0.02, 1.41]
**Subtotal (95% CI)**		**106**		**198**	**9.2%**	**0.47 [0.09, 2.42]**
Total events	4		16			
Heterogeneity: Tau² = 0.60; Chi² = 1.71, df = 1 (P = 0.19); I² = 41%						
Test for overall effect: Z = 0.90 (P = 0.37)						
**3.2.4 MM**						
29 Lima 2008 MM	11	90	12	139	11.3%	1.47 [0.62, 3.50]
**Subtotal (95% CI)**		**90**		**139**	**11.3%**	**1.47 [0.62, 3.50]**
Total events	11		12			
Heterogeneity: Not applicable						
Test for overall effect: Z = 0.88 (P = 0.38)						
**Total (95% CI)**		**783**		**1553**	**100.0%**	**1.69 [1.11, 2.56]**
Total events	102		136			
Heterogeneity: Tau² = 0.20; Chi² = 19.56, df = 12 (P = 0.08); I² = 39%						
Test for overall effect: Z = 2.47 (P = 0.01)						
Test for subgroup differences: Chi² = 4.34, df = 3 (P = 0.23), I² = 30.9%						

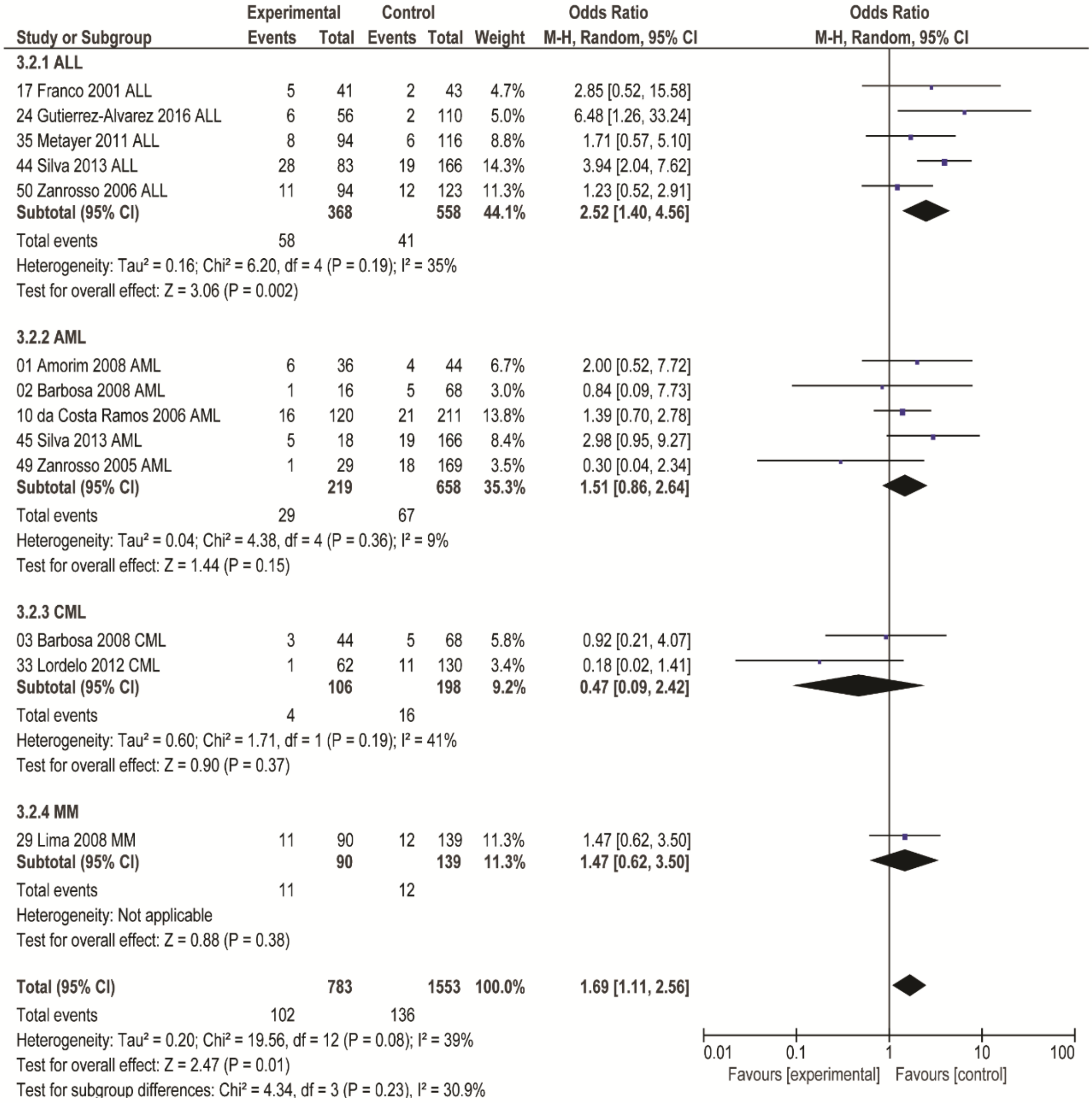

Figure S10 - A1298C Homozygous Model.
