# Supplementary material for: Differential effects of the methylenetetrahydrofolate reductase polymorphisms (C677T and A1298C) on hematological malignancies among Latinos: a meta-analysis
Source: Genet Mol Biol. 2019 Nov 14;42(3):549–59. doi: 10.1590/1678-4685-GMB-2018-0161 (PMC6905449; doi:10.1590/1678-4685-GMB-2018-0161)
Supplement: Supplementary file 14 [file 1415-4757-GMB-42-3-2018-0161-suppl11.pdf]

Supplementary Material to "Differential effects of the methylenetetrahydrofolate reductase polymorphisms (C677T and A1298C) on hematological malignancies among Latinos: a meta-analysis"

Study or Subgroup	Experimental Events	Experimental Total	Control Events	Control Total	Weight	Odds Ratio M-H, Random, 95% CI
**3.3.1 ALL**						
17 Franco 2001 ALL	35	71	30	71	6.3%	1.33 [0.69, 2.58]
24 Gutierrez-Alvarez 2016 ALL	20	70	44	152	6.7%	0.98 [0.53, 1.84]
35 Metayer 2011 ALL	68	154	68	178	9.8%	1.28 [0.82, 1.98]
44 Silva 2013 ALL	81	136	101	248	10.1%	2.14 [1.40, 3.28]
50 Zanrosso 2006 ALL	85	168	88	199	10.4%	1.29 [0.86, 1.95]
**Subtotal (95% CI)**		**599**		**848**	**43.2%**	**1.41 [1.09, 1.82]**
Total events	289		331			
Heterogeneity: Tau² = 0.02; Chi² = 5.38, df = 4 (P = 0.25); I² = 26%						
Test for overall effect: Z = 2.64 (P = 0.008)						
**3.3.2 AML**						
01 Amorim 2008 AML	20	50	20	60	5.0%	1.33 [0.61, 2.91]
02 Barbosa 2008 AML	12	27	37	100	4.3%	1.36 [0.58, 3.22]
10 da Costa Ramos 2006 AML	78	182	125	315	11.3%	1.14 [0.79, 1.65]
45 Silva 2013 AML	18	31	101	248	5.2%	2.02 [0.95, 4.30]
49 Zanrosso 2005 AML	14	42	95	246	5.9%	0.79 [0.40, 1.59]
**Subtotal (95% CI)**		**332**		**969**	**31.7%**	**1.20 [0.92, 1.57]**
Total events	142		378			
Heterogeneity: Tau² = 0.00; Chi² = 3.39, df = 4 (P = 0.49); I² = 0%						
Test for overall effect: Z = 1.35 (P = 0.18)						
**3.3.3 CML**						
03 Barbosa 2008 CML	26	67	37	100	6.6%	1.08 [0.57, 2.04]
33 Lordelo 2012 CML	44	105	154	273	9.5%	0.56 [0.35, 0.88]
**Subtotal (95% CI)**		**172**		**373**	**16.0%**	**0.75 [0.39, 1.42]**
Total events	70		191			
Heterogeneity: Tau² = 0.14; Chi² = 2.74, df = 1 (P = 0.10); I² = 63%						
Test for overall effect: Z = 0.89 (P = 0.37)						
**3.3.4 MM**						
29 Lima 2008 MM	44	123	61	188	9.0%	1.16 [0.72, 1.87]
**Subtotal (95% CI)**		**123**		**188**	**9.0%**	**1.16 [0.72, 1.87]**
Total events	44		61			
Heterogeneity: Not applicable						
Test for overall effect: Z = 0.61 (P = 0.54)						
**Total (95% CI)**		**1226**		**2378**	**100.0%**	**1.19 [0.97, 1.46]**
Total events	545		961			
Heterogeneity: Tau² = 0.06; Chi² = 22.21, df = 12 (P = 0.04); I² = 46%						
Test for overall effect: Z = 1.64 (P = 0.10)						
Test for subgroup differences: Chi² = 3.51, df = 3 (P = 0.32), I² = 14.5%						

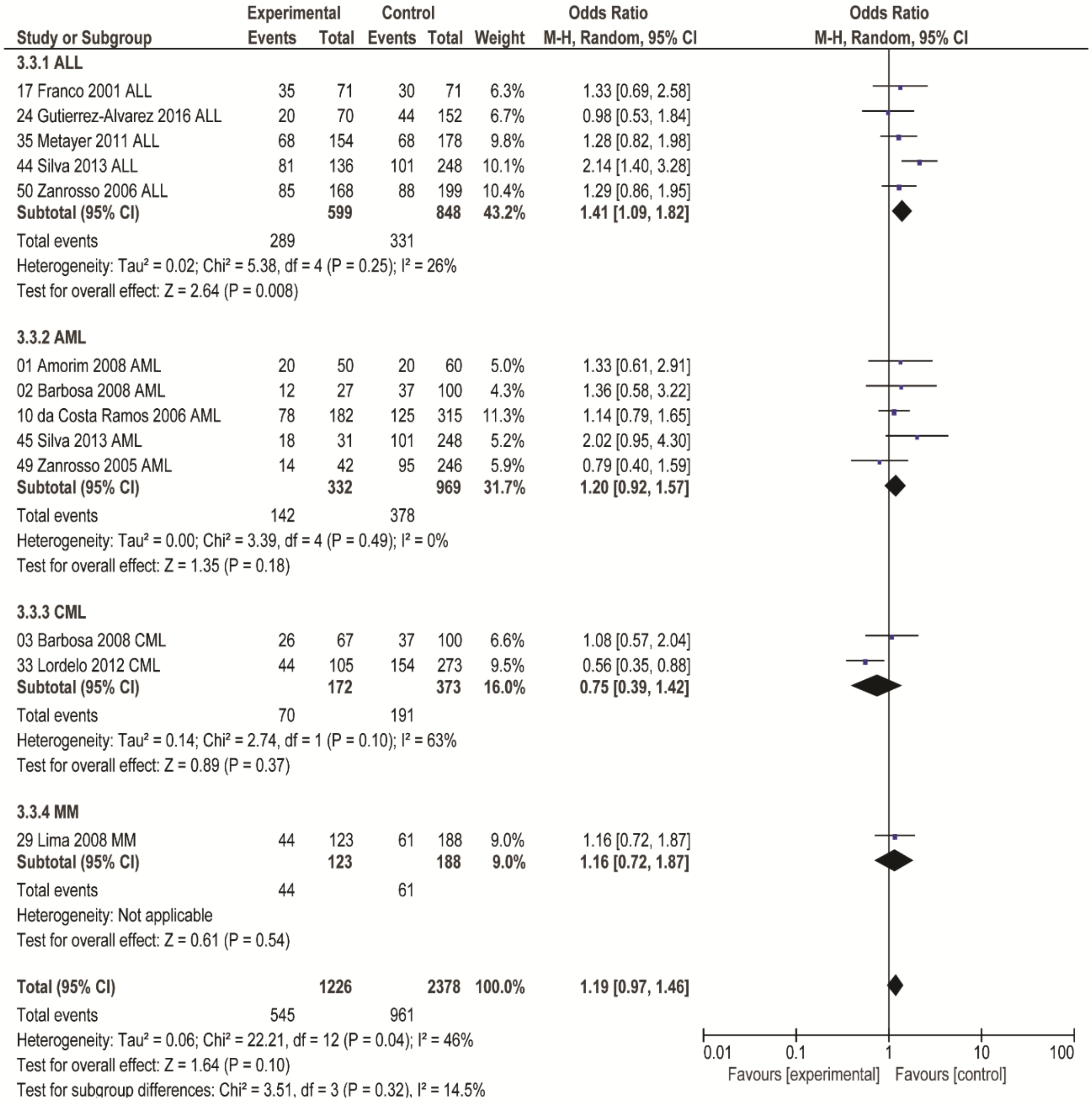

Figure S11 - A1298C Dominant Model.
