# Supplementary material for: Differential effects of the methylenetetrahydrofolate reductase polymorphisms (C677T and A1298C) on hematological malignancies among Latinos: a meta-analysis
Source: Genet Mol Biol. 2019 Nov 14;42(3):549–59. doi: 10.1590/1678-4685-GMB-2018-0161 (PMC6905449; doi:10.1590/1678-4685-GMB-2018-0161)
Supplement: Supplementary file 15 [file 1415-4757-GMB-42-3-2018-0161-suppl12.pdf]

Supplementary Material to "Differential effects of the methylenetetrahydrofolate reductase polymorphisms (C677T and A1298C) on hematological malignancies among Latinos: a meta-analysis"

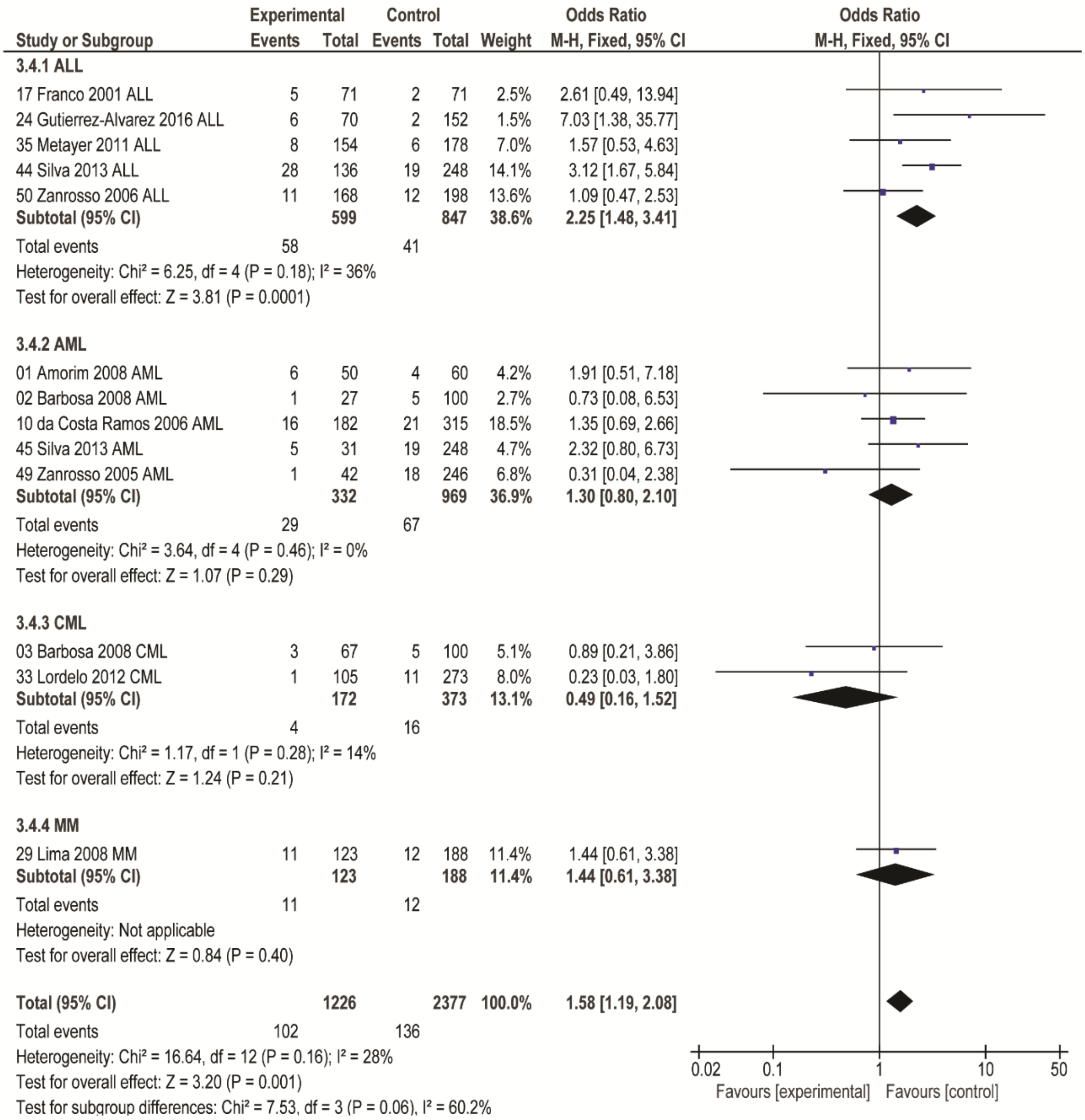

Figure S12 - A1298C Recessive Model.
