# Supplementary material for: Differential effects of the methylenetetrahydrofolate reductase polymorphisms (C677T and A1298C) on hematological malignancies among Latinos: a meta-analysis
Source: Genet Mol Biol. 2019 Nov 14;42(3):549–59. doi: 10.1590/1678-4685-GMB-2018-0161 (PMC6905449; doi:10.1590/1678-4685-GMB-2018-0161)
Supplement: Supplementary file 16 [file 1415-4757-GMB-42-3-2018-0161-suppl13.pdf]

Supplementary Material to "Differential effects of the methylenetetrahydrofolate reductase polymorphisms (C677T and A1298C) on hematological malignancies among Latinos: a meta-analysis"

Study or Subgroup	Experimental Events	Experimental Total	Control Events	Control Total	Weight	Odds Ratio M-H, Random, 95% CI
**3.5.1 ALL**						
17 Franco 2001 ALL	42	142	32	142	6.7%	1.44 [0.85, 2.46]
24 Gutierrez-Alvarez 2016 ALL	26	140	46	304	6.7%	1.28 [0.75, 2.17]
35 Metayer 2011 ALL	76	308	74	356	9.2%	1.25 [0.87, 1.80]
44 Silva 2013 ALL	109	272	120	496	10.1%	2.10 [1.52, 2.88]
50 Zanrosso 2006 ALL	96	336	100	398	9.9%	1.19 [0.86, 1.65]
**Subtotal (95% CI)**		**1198**		**1696**	**42.6%**	**1.44 [1.13, 1.83]**
Total events	349		372			
Heterogeneity: Tau² = 0.03; Chi² = 7.39, df = 4 (P = 0.12); I² = 46%						
Test for overall effect: Z = 2.96 (P = 0.003)						
**3.5.2 AML**						
01 Amorim 2008 AML	26	98	26	120	5.6%	1.31 [0.70, 2.44]
02 Barbosa 2008 AML	13	54	42	200	4.7%	1.19 [0.59, 2.43]
10 da Costa Ramos 2006 AML	94	364	146	630	10.4%	1.15 [0.86, 1.56]
45 Silva 2013 AML	23	62	120	496	6.4%	1.85 [1.06, 3.22]
49 Zanrosso 2005 AML	15	84	113	492	5.9%	0.73 [0.40, 1.32]
**Subtotal (95% CI)**		**662**		**1938**	**33.0%**	**1.19 [0.92, 1.55]**
Total events	171		447			
Heterogeneity: Tau² = 0.02; Chi² = 5.14, df = 4 (P = 0.27); I² = 22%						
Test for overall effect: Z = 1.33 (P = 0.19)						
**3.5.3 CML**						
03 Barbosa 2008 CML	29	134	42	200	6.7%	1.04 [0.61, 1.77]
33 Lordelo 2012 CML	45	210	165	546	9.0%	0.63 [0.43, 0.92]
**Subtotal (95% CI)**		**344**		**746**	**15.7%**	**0.78 [0.48, 1.27]**
Total events	74		207			
Heterogeneity: Tau² = 0.07; Chi² = 2.26, df = 1 (P = 0.13); I² = 56%						
Test for overall effect: Z = 1.01 (P = 0.31)						
**3.5.4 MM**						
29 Lima 2008 MM	55	246	73	376	8.7%	1.20 [0.81, 1.77]
**Subtotal (95% CI)**		**246**		**376**	**8.7%**	**1.20 [0.81, 1.77]**
Total events	55		73			
Heterogeneity: Not applicable						
Test for overall effect: Z = 0.89 (P = 0.38)						
**Total (95% CI)**		**2450**		**4756**	**100.0%**	**1.21 [1.00, 1.46]**
Total events	649		1099			
Heterogeneity: Tau² = 0.07; Chi² = 28.97, df = 12 (P = 0.004); I² = 59%						
Test for overall effect: Z = 1.94 (P = 0.05)						
Test for subgroup differences: Chi² = 5.11, df = 3 (P = 0.16), I² = 41.2%						

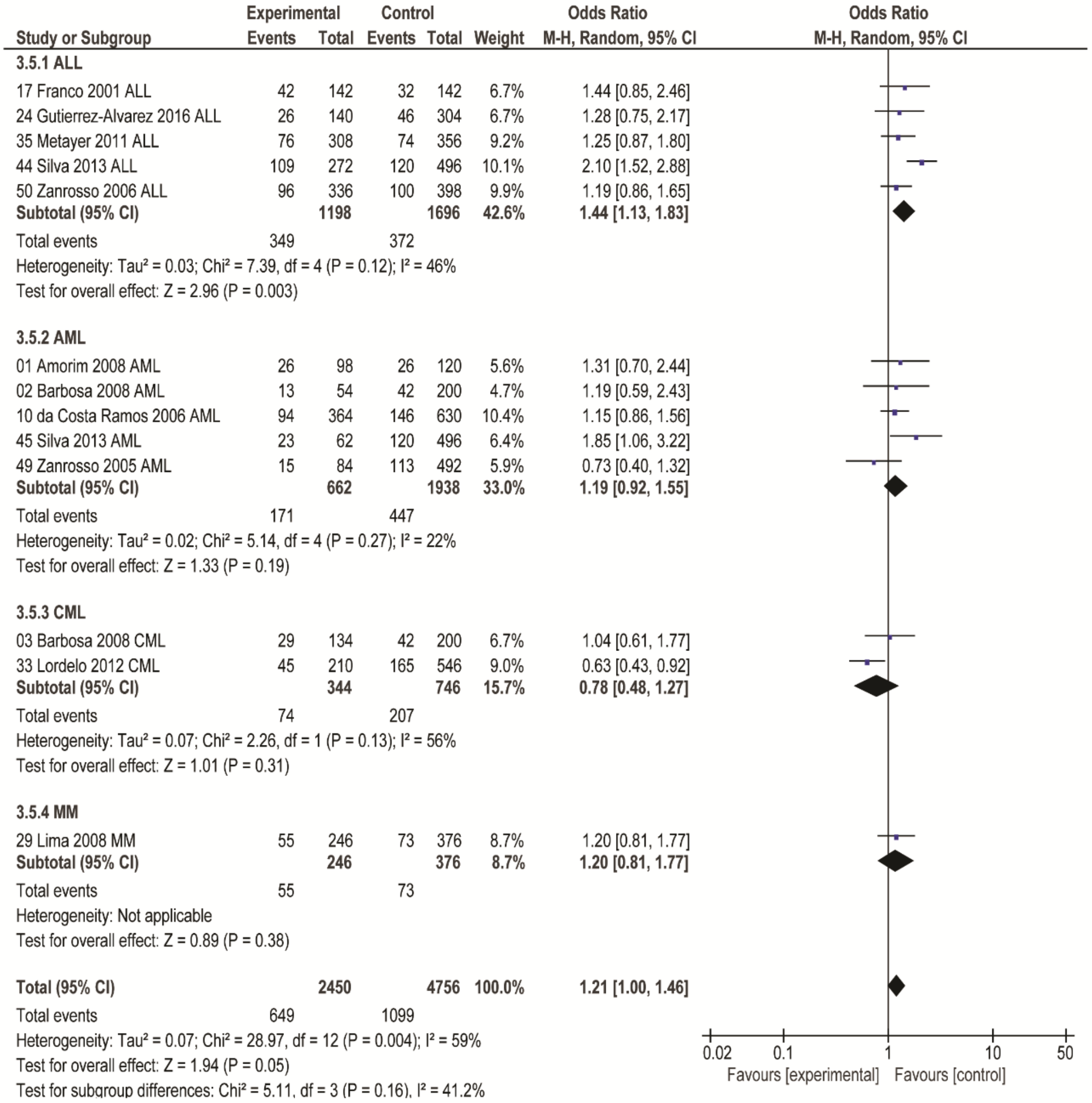

Figure S13 - A1298C Allelic Model.
